# Proteomic Characterization of Antibiotic Resistance, and Production of Antimicrobial and Virulence Factors in *Streptococcus* Species Associated with Bovine Mastitis. Could Enzybiotics Represent Novel Therapeutic Agents Against These Pathogens?

**DOI:** 10.3390/antibiotics9060302

**Published:** 2020-06-04

**Authors:** Ana G. Abril, Mónica Carrera, Karola Böhme, Jorge Barros-Velázquez, José-Luis R. Rama, Pilar Calo-Mata, Angeles Sánchez-Pérez, Tomás G. Villa

**Affiliations:** 1 Departamento de Microbiología y Parasitología, Facultad de Farmacia, Campus Sur 15782, Universidad de Santiago de Compostela, 15705 Santiago de Compostela, Spain; anagonzalezabril@hotmail.com (A.G.A.); joserodrama@gmail.com (J.-L.R.R.); 2 Marine Research Institute (IIM), Spanish National Research Council (CSIC), Eduardo Cabello 6, 36208 Vigo, Pontevedra, Spain; mcarrera@iim.csic.es; 3 Agroalimentary Technological Center of Lugo, Montirón 154, 27002 Lugo, Spain; karolaboehme@gmx.de; 4 Departamento de Química Analítica, Nutrición y Bromatología, Area de Tecnología de los Alimentos, Facultad de Veterinaria, Campus Lugo, Universidad de Santiago de Compostela, 27002 Lugo, Spain; jorge.barros@usc.es (J.B.-V.); p.calo.mata@gmail.com (P.C.-M.); 5 Sydney School of Veterinary Science, Faculty of Science, University of Sydney, Sydney, NSW 2006, Australia; angelines2085@icloud.com

**Keywords:** LC–ESI–MS/MS, proteomics, mass spectrometry, characterization of antibiotic resistance peptides, lantibiotic production, virulence factors, *Streptococcus* spp.

## Abstract

*Streptococcus* spp. are major mastitis pathogens present in dairy products, which produce a variety of virulence factors that are involved in streptococcal pathogenicity. These include neuraminidase, pyrogenic exotoxin, and M protein, and in addition they might produce bacteriocins and antibiotic-resistance proteins. Unjustifiable misuse of antimicrobials has led to an increase in antibiotic-resistant bacteria present in foodstuffs. Identification of the mastitis-causing bacterial strain, as well as determining its antibiotic resistance and sensitivity is crucial for effective therapy. The present work focused on the LC–ESI–MS/MS (liquid chromatography–electrospray ionization tandem mass spectrometry) analysis of tryptic digestion peptides from mastitis-causing *Streptococcus* spp. isolated from milk. A total of 2706 non-redundant peptides belonging to 2510 proteins was identified and analyzed. Among them, 168 peptides were determined, representing proteins that act as virulence factors, toxins, anti-toxins, provide resistance to antibiotics that are associated with the production of lantibiotic-related compounds, or play a role in the resistance to toxic substances. Protein comparisons with the NCBI database allowed the identification of 134 peptides as specific to *Streptococcus* spp., while two peptides (EATGNQNISPNLTISNAQLNLEDKNK and DLWC*NM*IIAAK) were found to be species-specific to *Streptococcus dysgalactiae*. This proteomic repository might be useful for further studies and research work, as well as for the development of new therapeutics for the mastitis-causing *Streptococcus* strains.

## 1. Introduction

*Streptococcus* spp. are major mastitis pathogens present in dairy products [1]; they cause considerable economic losses and also affect human health [2,3]. Streptococcal species, such as *S. agalactiae*, *S. canis, S. dysgalactiae*, and *S. uberis*, are the main species involved in clinical and subclinical bovine mastitis [3,4]. Additional species were identified as minor mastitis-causing pathogens, these include *S. gallolyticus* [5] and *S. parauberis* [6].

*Streptococcus* produce a variety of virulence factors that are involved in streptococcal pathogenicity, as well as playing a role in their anti-phagocytic activity and their strong adsorption on surfaces and cells [7]. These factors include neuraminidase, pyrogenic exotoxin, and M protein [7]; in addition, *Streptococcus* spp. can produce bacteriocins, such as lantibiotics [8,9,10,11] and antibiotic resistance proteins. As a result, these microorganisms are responsible for up to 30% of bovine mastitis cases [7].

Mastitis prevention is important and involves giving the cows the right nutritional balance, lack of stress in the animals and adequate farm sanitation. Nevertheless, it is not always possible to fully avert this disease, even when using antimicrobial therapy, which is the most common treatment for mastitis. Therefore, identification of the mastitis-causing bacterial strain, as well as determining its antibiotic resistance and sensitivity, is crucial for an effective therapy. Classical strain identification methods are cumbersome and time-consuming, hence new techniques must be implemented for a quick and unequivocal bacterial identification. This is further complicated by the overuse and misuse of antibiotics, a common practice in the dairy industry, and in animal farms in general; it is currently estimated that 56% of farmers worldwide use non-prescribed antibiotics [12]. This unjustifiable misuse of antimicrobials has led to an increase in antibiotic-resistant bacteria present in foodstuffs; these bacteria represent a considerable hazard to humans, making it essential to tightly regulate the use of antibiotics [13].

Conventional antibiotic therapy is currently often unsuccessful and its widespread use also leads to antibiotic resistance; new strategies must involve novel disease therapies, such as enzybiotics, to replace obsolete antibiotic treatment. The term enzybiotics is a neologism formed by the words “enzyme” and “antibiotic”, which was coined Nelson et al. in 2001. These researchers, from The Rockefeller University, used the new name to describe bacteriophage-encoded endolysins that could be used as an alternative to antibiotic therapy to combat bacterial infections, in particular those caused by Gram-positive microorganisms [14]. This novel therapeutic approach includes the use of both bacteriophages and bacteriocins, such as nisin [15,16], which display a broad antimicrobial spectrum against Gram-positive bacteria [13], as well as purified bacterial and bacteriophage lysins. The term enzybiotics currently has a more extensive meaning, including antifungal enzymes and, even, antiviral compounds.

*Streptococcus* spp. and *Staphylococcus* spp. species are, in general, sensitive to penicillin, and this compound remains the drug of first choice to combat bovine mastitis; but unfortunately, both genera of pathogenic bacteria are quickly developing resistance to this antibiotic [17]. This makes it imperative to expedite the search for new antimicrobials and technologies that can be used for therapy in veterinary medicine.

The use of techniques such as polymerase chain reaction (PCR), random amplified polymorphic DNA (RAPD), and whole genome sequencing has permitted the identification of mastitis-causing *Streptococcus* species that is highly resistant to antimicrobials, which, in addition, contain strong virulence genes [7,18,19]. DNA analyses of the genes responsible for antimicrobial resistance, and for virulence, provide essential information on the mechanisms involved in these processes. In addition, proteome analyses identify the proteins are being produced in a given environment, allowing effective and rapid proteomic fingerprinting for prompt and precise microbial identification. Techniques like liquid chromatography–electrospray ionization–tandem mass spectrometry (LC–ESI–MS/MS) were successfully used to specifically identify pathogenic bacterial strains [20,21,22].

Here, we report the use of LC–ESI–MS/MS to differentiate the proteomics of both antibiotic resistance and the production of antimicrobials and other virulence factors, with the aim to characterize and identify the different *Streptococcus* species associated with mastitis.

## 2. Results

### 2.1. Streptococcus spp. Proteomics Data Repository

Fourteen different *Streptococcus* spp. strains were studied, their protein mixtures were digested with trypsin, and the resulting peptides were analyzed by LC–ESI–MS/MS. This procedure identified a total of 2706 non-redundant peptides, corresponding to 2510 different annotated proteins, from the *Streptococcus* spp. strains examined (see complete non-redundant dataset in Table S1). Virulence factors were identified with the “Virulence Factors of Pathogenic Bacteria Database” (VFDB; http://www.mgc.ac.cn/VFs/), while antibiotic resistance proteins were determined using the “Comprehensive Antibiotic Resistance Database” (https://card.mcmaster.ca/). Additional virulence factors were identified by comparison with the published proteins [23,24].

From the 2706 non-redundant peptides obtained above, 168 were identified as virulence factors. This group include proteins involved in eukaryotic cell colonization and immune evasion, such as the M protein, which is specific to the *Streptococcus* spp.; additional compounds encompass toxins and antitoxins, proteins involved antibiotic resistance, and lantibiotics. All 168 virulent factors identified are summarized in Table S2.

In addition, the 168 virulence factors were analyzed by the Basic Local Alignment Tool for proteins (BLASTp), which used an algorithm to compare the protein sequences and find the regions of homology within amino acid sequences in the NCBI database [25]. Some of the BLASTp searches included the term *Streptococcaceae*, to identify peptides specific to the *Streptococcus* species, with 100% homology to proteins in the NCBI database. Table 1 summarizes the 134 virulence factors identified as specific to *Streptococcus* spp., classified by their main role as—toxins and antitoxins, colonization and immune evasion factors, antibiotic resistance peptides, proteins involved in resistance to toxic substances, compounds involved in antimicrobial production, and ABC transporters and other transporters associated with virulence factors. In fact, this article represents the first report for 166 of these virulence factor peptides, as they have not been previously described for the particular strains analyzed here. Only two peptides identified in this study (DLWC*NM*IIAAK and EATGNQNISPNLTISNAQLNLEDKNK) were previously described in *S. dysgalactiae* strains.

As seen in Table 1, the number of virulence factors, discovered by LC–ESI–MS/MS analyses, which were specific to *Streptococcus*, varied according to the strain examined, as did the number of those factors that were not specific to the genus.

### 2.2. Proteins Involved in Bacterial Resistance to Antibiotics or Other Toxic Substances

Our study identified sixteen peptides, present in *Streptococcus* spp. strains, which are involved in resistance to either antibiotics or toxic substances (Table S2); according to the NCBI database, thirteen of these peptides were specific to *Streptococcus* spp. (Table 1). Thirteen out of the sixteen proteins were associated with antibiotic resistance; they involved three peptides identified as *Streptococcus* spp.-specific, belonging to the multiple antibiotic resistance repressor (MarR) family of proteins, and present in strains ST2, ST3, and ST4. MarR acts as a regulator for proteins involved in resistance against several antibiotics [26]. In addition, a MurM peptide was identified for the ST9 strain; this protein played a role in the modification of cellular wall muropeptides, therefore, increasing both penicillin and cephalosporin resistance [27]. Furthermore, two peptides, from strains ST8 and ST6, were characterized as beta-lactamase class A. Moreover, a peptide identified in the ST1 strain represented an M56 type protein; the M56 family of proteins are metallopeptidases involved in beta-lactamase transduction [28]. A TipAS protein was found in the ST6 strain; TipAS are antibiotic-recognition domain containing proteins that confer resistance to thiostrepton and similar antimicrobial compounds [29]. ST3 strains also contained an additional peptide, corresponding to a protein involved in the microbial response to antibiotics, which attacks the bacterial cell wall. This protein recognizes perturbations in the cell envelope, caused by antibiotics such as bacitracin and vancomycin, which interfere with the lipid II metabolism and the undecaprenyl cycle [30]. Another peptide, also identified in the ST3 strain, corresponds to a streptomycin adenylyltransferase, a protein that mediates bacterial resistance to the antibiotics streptomycin and spectinomycin [31]. Other peptides characterized included two penicillin binding proteins, discovered in ST1 and ST12, which have an affinity for the antibiotic [32]; while one peptide belonging to the Glyoxalase/Bleomycin resistance protein was identified in ST14.

The proteins involved in resistance to toxic substances included three peptides, found in the ST4 strain, which represent the toxic anion resistance protein TelA [33] involved in tellurite resistance; as well as two mercury resistance proteins, MerA and MerR, present in ST1 and ST9 strains, respectively. MerR proteins are prokaryotic metal ion sensing regulators, which allow transcription of several Mer genes to produce mercury resistance phenotypes [34]; while MerA increases Hg^2+^ resistance in bacteria, by volatilizing mercury as Hg^0^ [35].

### 2.3. Proteins Involved in Bacteriocin Production

Five of the peptides identified by LC–ESI–MS/MS analyses represent either antimicrobials or proteins involved in their production (Table S2). According to the NCBI database, these peptides were specific for *Streptococcus* spp. and either constitute bacteriocins or modified bacteriocins, such as lantibiotics (Table 1).

Two bacteriocin-related peptides were discovered in the ST2 and ST3 strains; in addition, the ST14 strain contain a bacteriocin-associated integral membrane protein. Furthermore, strains ST12 and ST4 also produce similar peptides, with homology to lantibiotic biosynthesis proteins such as LanM and LanT, which are involved in post-translational modifications and processing [9], respectively. Additional peptides were identified as part of the lantibiotic transporter group (see Section 2.6 below).

### 2.4. Proteins Involved in Host Colonization and Immune Evasion

This study identified 81 proteins, obtained from the different bacterial strains, which are present in the proteomic repository of the *Streptococcus* spp.; these are involved in colonization and immune evasion (Table S2) and, according to the NCBI database, 64 of these peptides are specific to the *Streptococcus* spp. (Table 1).

From the 81 peptides associated with bacterial pathogenicity, five N-acetylmuramoyl-L-alanine amidases were identified in strains ST1, ST8, ST10, ST7, and ST14. These proteins are autolysins that play a role in bacterial adherence to eukaryotic cells [19]. Two additional peptides, representing a bifunctional autolysin and a lysin, were characterized in strains ST6 and ST8; while two peptides, from strains ST4 and ST10, constitute lysozymes.

The LysM domain is a general peptidoglycan-binding module, originally discovered in enzymes that degrade bacterial cell walls, but that are also present in many other bacterial and eukaryotic proteins involved in pathogenesis [36]. The additional identified peptides play a role in virulence, such as a TcpC transposase-detected in the ST9 strain; this protein is part of the Tcp conjugation system, involved in the conjugative transfer of large virulence plasmids in pathogenic bacteria [37]. Moreover, eleven peptides, obtained from the strains ST3, ST5, ST7, ST9, ST8, and ST14, belong to the CLp ATases; these include four CLpX ATases and two CLpC ATases. CLp proteins are composed by a CLp ATase and a peptidase, the latter degrades the proteins involved in the modulation of virulence factors. In fact, CLpX modulates the proteolytic activity of specific enzymes, as well as biofilm formation in *Streptococcus mutans*; while CLpC is involved in *Streptococcus pneumoniae* virulence [38,39]. Further discoveries include four sialidases, from strains ST3, ST13, ST14, and ST6, and a neuraminidase A, present in the ST6 strain. Sialidases and neuraminidases are bacterial hydrolytic enzymes that modify and eliminate sialic acid from glycoproteins, glucolipids, gangliosides, and polysaccharides; this helps expose the adhesins on the surface of the bacterial cell [40,41].

Two additional peptides, from strains ST10 and ST2, belong to the choline-binding protein group (Cbp); these proteins are composed of two modules, the first module displays biological activity, while the second binds a choline residue on the bacterial cell wall. These proteins were identified as virulence factors and are also present in bacteriophages; peptide EGSTWYYLKGSGAM*ATGWATANGQWSYFEK contains an N-acetylmuramoyl-L-alanine amidase in its sequence. Some Cbp proteins include an N-acetylmuramoyl-L-alanine amidase in their biological module, which is involved in peptidoglycan releasing, pro-inflammatory teichoic acid formation, as well as in cell division and bacterial colonization [42]. Two further Cpb peptides were obtained from the strains ST4 and ST7. These represent specific Cpb proteins (PspA) that are involved in lactoferrin binding and in inhibition of the inactivation factor [42]. Some PspA sequences are homologous to glucosyltransferases, as is the case for peptide TEQVLLTEAVQQVQR.

Three peptides were identified, in strains ST4 and ST2, as IgA1 proteases, metallopeptidases that bind mammal IgA1 [43]; while two additional peptides were characterized as C5A peptidases, in strains ST3, ST13, and ST12. These proteins encompass a sortase A, and are involved in C5a complement factor inactivation, resulting in leukocyte stimulation [23]. Sortases are polypeptides that covalently attach secreted proteins to their cell wall to assemble pili, they play a key role in the infection process and represent potential drug targets [44]; two of the peptides identified corresponded to the sortase A group; while one sortase B and one sortase C, were also characterized.

Four peptides, corresponding to M protein, were discovered in strains ST1, ST13, and ST14; the M protein is characteristic of streptococci cell walls and, when released into the bloodstream, it forms complexes with neutrophils and monocytes that activate the inflammatory response [23]. Mga, on the other hand, is a virulence factor, as is the case for the M protein and the C5a peptidase [45]; three Mga proteins were identified from the strains ST9, ST14, and ST7.

Acetylase and deacetylase enzymes were also among the proteins identified in the *Streptococcus* spp. strains; these included O-acetylase (OafA) and Peptidoglycan-N-acetylglucosamine deacetylase, which play a role in peptidoglycan deacetylation, avoiding eukaryotic lysozyme recognition during infection [46,47]. One peptide representing an O-acetylase (OafA) was identified in ST5, while a peptidoglycan-N-acetylglucosamine deacetylase was present in strain ST6. Furthermore, two superoxide dismutase enzymes (SOD) were characterized from the strains ST10 and ST14; after infection, macrophages and neutrophils produce toxic superoxides to fight incoming bacteria, and SOD is a metalloenzyme that decomposes the superoxides and promotes bacterial invasion [48].

The type II secretion (T2S) system is involved in pathogenic processes, including the death of host cells, bacterial adherence to host surfaces, growth within host cells, and innate immunity suppression [49]; one T2S protein was identified in the ST4 strain. Type VII protein secretion (ESS) is a specialized system that facilitates secretion of extracellular proteins across the cytoplasmic membrane; it plays a role in establishing host infection, which was associated with virulence in *S. aureus* [50]. Two peptides belonging to the type VII secretion EssB protein were characterized in strains ST2 and ST8, while one type VII secretion EsaA peptide was identified in ST2. Capsular polysaccharides (CPS) contribute to pathogenesis by inhibiting the entrapment of pneumococci in neutrophil extracellular traps; the cpsABCD locus is involved in the modulation of CPS biosynthesis [51], while the CapD protein is required for type I CPS biosynthesis [52]. Two CpsC peptides were isolated from strains ST1 and ST5, while a CpsB protein was identified for ST6; in addition, a CapD protein was obtained from the ST13 strain.

Adhesion pili are virulence factors present on the surface of bacteria; they are usually required for biofilm formation, enabling the bacteria to bind and adhere to target cells [53]. Some pili-associated proteins were identified from the *Streptococcus* strains analyzed, these included peptides corresponding to an accessory pilus subunit denominated fimbrial low-molecular weight protein (Flp); pilus assembly protein (CpaB) was discovered in strains ST3 and ST23, while a FimC protein was characterized in the ST8 strain. Accessory pilus subunits are adhesins found on the bacterial cell surface that are important for binding to host cells; Flp polypeptides, on the other hand, are pili biogenesis proteins [54], while FimC acts as a chaperone and is required for the assembly of type-1 pili [55].

The agglutinin receptor mediates agglutinin aggregation; in oral streptococci, the interaction between the sialic acid residues of salivary agglutinin (SAG) and its receptor, modulates bacterial colonization of the oral tissue [56]. Four agglutinin receptor peptides were identified in strains ST12, ST3, ST7, and ST9. Furthermore, a bacillolysin-type peptide was discovered in the ST2 strain; bacillolysin is a metalloproteinase, produced by *Bacillus* strains, which affects blood coagulation and the fibrinolytic system [57].

Adhesin peptides were identified in three strains, ST1, ST8, and ST1; these proteins include P1 adhesin (also called antigen I/II or PAc), located on the cell surface and produced by the cariogenic bacterium *Streptococcus mutans*, which mediate sucrose-independent adhesion to tooth surfaces [58]. A CppA polypeptide was also characterized, it represents a putative C3-glycoprotein degrading proteinase involved in pathogenicity [59]. In addition, a peptide belonging to the LytR family of transcriptional regulator proteins was identified in the ST5 strain; the LytSR two-component regulatory system controls the activity of murein hydrolase, stationary-phase survival, antibiotic tolerance, and biofilm formation [60].

Three peptides corresponding to alkaline shock protein 23 (Asp23) were identified in the strains ST2, ST12, and ST14; the Asp23 family proteins are characteristic of Gram-positive bacteria. They are functionally linked to lipid metabolism in *Bacillus subtilis*, while in *Streptococcus agalactiae* they play a role in survival at low pH and during nutrient limitation; in *Enterococcus faecalis* they control cell morphology, whereas they are involved in nutrient sensing in *Streptococcus pneumoniae* [61]. A peptide in the ST14 strain was identified as general stress protein 17, associated with heat shock, salt stress, and oxidative stress, as well as glucose and oxygen limitation.

Siderophores are small molecules specialized in iron-acquisition; the production of siderophores can give a pathogen an advantage in its competition with other bacteria, as well as modulating host cellular pathways during infection [62]. Equibactin is a siderophore reported only in *Streptococcus equi*; this article represents the first time that this peptide was identified in a different species, as here we report its presence in the ST9 strain of *S. dysgalactiae* [63]. Finally, two virulence-associated proteins were identified in strains ST3 and ST14.

### 2.5. Proteins Involved in Bacterial Toxicity

Amino acid sequence comparisons, between the peptides obtained from the different bacterial strains studied and the *Streptococcus* spp. proteomic repository, identified seven proteins involved in bacterial toxicity (Table S2). According to the NCBI database, six of these peptides are specific to the *Streptococcus* spp. These include a pyrogenic exotoxin, toxins, and antitoxins from the *relBE* and *yefM-yoeB* operons, a Beta-class phenol-soluble modulin, and a Doc (death-on-curing) family toxin (Table 1). The ST1 strain contained a RelE toxin protein, while a member of the antitoxin RelB was identified in the ST3 strain. The *relBE* operon plays a role in nutrient stress, in both bacteria and archaea, and contains toxin–antitoxin modules that are subjected to autoregulation. Toxin ReIE is a stable protein, while antitoxin RelB is easily hydrolyzed by the Lon protein; in nutrient-depleted environments, antitoxin RelB transcription decreases, hence the protein stops acting as a repressor, allowing production and release of the RelE toxin.

Peptides isolated from the ST2 strain were identified as YefM antitoxin and its corresponding toxin YoeB; YefM is an antitoxin belonging to the toxin–antitoxin type II system, encoded by the *yefM-yoeB* operon. This protein is involved in *E. coli* and *Streptococcus* spp. toxicity, as well as in biofilm formation in *S. pneumoniae* [64]; although biofilm formation also depends on the expression of the Hha protein [65,66]. In addition, overexpression of YoeB, in the absence of YefM, arrests bacterial growth.

Strains ST11 and ST12 contain a protein corresponding to the pyrogenic SpeK exotoxin; SpeK exotoxins are involved in shock toxic syndrome in *Streptococcus* spp., acting as superantigens and facilitating tissue evasion and pro-inflammatory substance liberation [67], which can finally result in multiorgan failure [68,69].

The LC–ESI–MS/MS analyses also revealed the presence of a beta-class phenol-soluble modulin in the ST3 strain; modulin is a toxin family protein that contributes to biofilm development, it also generates cellular lysis of red and white blood cells and stimulates the inflammatory responses [70].

An additional peptide was identified as a Doc family toxin, in the ST9 strain; the Doc toxin belonged to the type II toxin–antitoxin system and is involved in the repression of transcription by DNA binding, this kinase is also present in *Archaea* and in bacteriophages [71].

### 2.6. Transporters Associated to Virulence Factors

The ABC transporters that represent virulence factors, were identified in 47 of the peptides obtained. Furthermore, an additional 10 peptides, corresponding to different types of transporters, were also found to facilitate virulence strategies (Table S2). According to the NCBI database, 81% (46 out of 57) of these peptides are specific to the *Streptococcus* spp. (Table 1).

**Figure 1:**
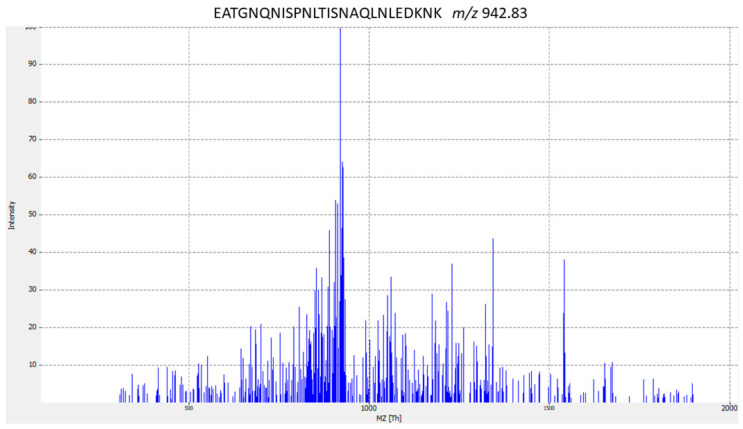
MS/MS spectrum for EATGNQNISPNLTISNAQLNLEDKNK *S. dysgalactiae-* specific peptide biomarker. The corresponding peptides were tested for specificity using the BLASTp algorithm.

Pathogenic bacteria display nutrient deprivation strategies to obtain dietary elements essential for growth, such as metal ions, amino acids, vitamins, and oligopeptides, and respond to hardship by releasing stress proteins and via immune evasion mechanisms. Several ABC-type transporters are involved in virulence, playing a role in bacterial propagation during infection [72,73,74,75]. The LC–ESI–MS/MS analyses carried out in *Streptococcus* spp. identified, in many of the analyzed strains, a series of peptides corresponding to the proteins required for the uptake of metals, such as zinc, copper, ferrous iron, cobalt, and nickel. Three peptides correspond to the FeoABC transporter system; one of these peptides containing the sequence EATGNQNISPNLTISNAQLNLEDKNK is specific to *S. dysgalactiae*, according to the NCBI database. Figure 1 shows the MS/MS spectrum for peptide biomarker that is species-specific for *S. dysgalactiae*.

Additional ABC transporters include three peptides identified in the strains ST1, ST14, and ST2; these are ABC transporters that carry bacitracin and are involved in bacitracin resistance [76]. A second group of these ABC-type transporters, found in several of the strains analyzed, are the multidrug ABC transporters; these play a role in antibiotic resistance and allow pathogenic bacteria to circumvent many currently used antimicrobial therapies [77]. Ten multidrug ABC transporters were identified in this study; five peptides represent bacteriocin class I and class II ABC transporters. Three peptides, present in strains ST4, ST9, and ST12, correspond to bacteriocin ABC transporter; while two peptides were identified, in the ST4 and ST9 strains, as lantibiotic ABC transporters, and one of them is specific to mutacin.

Further ABC-type transporters and related bacterial virulence factors, discovered in many analyzed strains, include the glutamine ABC transporter and glycine/betaine ABC transporter [73]; in addition, a peptide corresponding to an ABC transporter was identified in the ST14 strain, and a Choline ABC transporter in ST13. A polypeptide representing the macrolide ABC transporter (MacB) was isolated from the ST14 strain; the MacB protein confers resistance to macrolide drugs, and they have been described to play a role in both colistin and bacitracin resistance [78].

**Figure 2:**
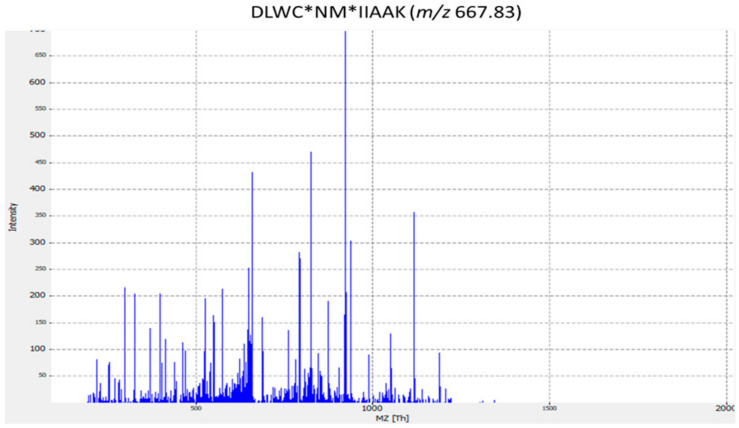
MS/MS spectrum for DLWC*NM*IIAAK *Streptococcus dysgalactiae*-specific peptide biomarker. The corresponding peptides were tested for specificity using the BLASTp algorithm.

Eleven peptides were identified as members of the group of transporters playing a role in ‘resistance to toxic substances’; these includes the manganese transport protein MntH, discovered in strains ST8 and ST2. Three peptides belonging to the HlyC/CorC transporter family, found in strains ST2, ST6, and ST8, were involved in magnesium and cobalt efflux. Furthermore, one peptide from the ST4 strain was identified as a metal-binding protein; this protein played a role in bacterial resistance to toxic metals, such as lead and cadmium [79]. A further three peptides were characterized as multidrug transporters; two of these proteins belonged to the MFS (major facilitator superfamily) transporter, one of the largest groups of solute transporters [80]. The third peptide corresponded to a protein of the multidrug and toxic compound extrusion (MATE), and was discovered in the ST9 strain; MATE proteins act as exporters of cationic drugs, such as norfloxacin and ethidium bromide, through H^+^ or Na^+^ exchange [81]. The remaining peptide, identified in the ST14 strain, represented a lantibiotic MFS transporter; this protein, containing the amino acid sequence DLWC*NM*IIAAK, is specific for *S. dysgalactiae*, according to the NCBI database. Figure 2 displays the MS/MS spectrum for this species-specific peptide biomarker of *S. dysgalactiae*.

## 3. Discussion

This paper describes the analysis and characterization of peptides isolated from fourteen protein extracts, corresponding to fourteen bacterial strains cultivated from the mastitis-causing *Streptococcus* species isolated from raw milk. LC–ESI–MS/MS analyses identified 168 peptides representing proteins that act as virulence factors, toxins, anti-toxins, provide resistance to antibiotics, are associated with the production of lantibiotic-related compounds, or play a role in the bacterial resistance to toxic substances. Protein comparisons with the NCBI database allowed the identification of 134 peptides as specific to *Streptococcus* spp.; while two peptides (EATGNQNISPNLTISNAQLNLEDKNK and DLWC*NM*IIAAK) were found to be species-specific to *S. dysgalactiae*. The pathogenic bacterial strains causing mastitis were identified by detection and characterization of specific diagnostic peptides [19,21,22,82,83,84], using proteomic techniques involving matrix-assisted laser desorption/ionization time of flight mass spectrometry (MALDI–TOF MS) and LC–ESI–MS/MS instruments. The precise method implemented in this study represent a useful framework for future research and analysis into pathogenic bacteria, as it offers advantages over previous approaches and allows direct identification of peptides, without the need for genomic sequencing and analysis.

The *Streptococcus* strains studied contain many peptides involved in the antibiotic resistance to penicillin, mediated by beta-lactamases and including beta-lactamase class A, MurM, MarA, M56, and multidrug ABC transporters [27]. The prevalence of proteins involved in penicillin resistance in the mastitis-causing bacterial strains is most concerning, as this compound is the principal antimicrobial agent used in the treatment of bovine mastitis caused by either *Streptococcus* spp. or *Staphylococcus* spp. In fact, penicillin resistance is increasing at a high rate all over the world [15], making it essential that pathogenic bacterial strains are quickly identified and their antibiotic resistances characterized, in order to provide the appropriate antimicrobial treatment. This article represents a proof of concept that bacterial identification and characterization can be quickly achieved by the use of LC–ESI–MS/MS. In addition, the approach used here to identify specific peptides characteristic to different microbial species and strains, could have wide applications in human health. Furthermore, the characterization of pathogenic factors, as proposed in the present study, would provide alternative treatments to classic antibiotics; these include the development of vaccines based on these virulence factor peptides. In addition, these peptides could represent future diagnostic tools for human pathogens, such as streptococci, which can invade cells and conceal themselves intracellularly. This is the case for some kidney and heart infections, such as acute glomerulonephritis and rheumatic fever, respectively, where specific bacterial peptides could be detected in either blood samples or other body fluids. This proteomic repository (Table S2) might be useful for further studies and researchers, as well as for the development of new therapeutics for mastitis-causing *Streptococcus* strains.

There is an urgent need for novel therapies to both treat and prevent mastitis [16]. Enzybiotics derived from endolysins and bacteriophage-encoded enzymes constitute a very promising class of compounds, as they display high specificity and, hence, low probability for the development of bacterial resistance [15]. Several recent studies have demonstrated, in a variety of animal models, the effectiveness of enzybiotics in the treatment of different mastitis-causing pathogen bacteria [85,86,87,88,89,90].

Furthermore, several bacteriocins are active against antibiotic resistant bacterial strains and could be used, either independently or in combination with other antimicrobials (antibiotics or enzybiotics), to prevent biofilm formation, a common characteristic of mastitis-causing pathogens. Nisin is a polycyclic antibacterial peptide, currently licensed as a food biopreservative; this antibacterial is as effective as conventional chemical treatments, such as iodine and chlorhexidine, against bovine mastitis-associated bacteria, including *S. aureus, S. agalactiae, S. dysgalactiae, Klebsiella pneumonia*, and *E. coli*. Another effective mastitis treatment is to use lacticin as a teat dip, a treatment shown to successfully reduce mastitis pathogens on the teats of lactating cows. In addition, staphylococcal, *Bacillus* sp. and other LAB bacteriocins can also efficiently combat bovine mastitis pathogens [16].

An alternative could be the treatment of bovine mastitis with bacteriophage therapy [90], as several temperate phage mixtures have proved to be more effective than using a single temperate phage for inhibiting *S. aureus.* Indeed, virulent phages, such as SPW and SA, are active against the bovine mastitis-causing *S. aureus*; while JX01 exhibit lytic activity against clinical *S. agalactiae* strains, a major bovine mastitis pathogen. Furthermore, SAJK-IND and MSP phages display specific lytic activity against several *S. aureus* strains isolated from milk produced by cows suffering from mastitis [85]. A phage cocktail, containing vBSM-A1 and vBSP-A2, was described as an effective treatment for mice with induced mastitis [86]. Endolysins, produced by bacteriophages, can successfully lyse the pathogen outer membrane; while Phi11 is capable of lysing mastitis-causing staphylococcal pathogens [16]. Schmelcher and co-workers [87] demonstrated the action of a two chimeric endolysins in an *S. aureus*-induced mastitis mouse model; the chimeric proteins were created by combining the streptococcal kSA2 endolysin endopeptidase domain with the staphylococcal cell wall binding domains from either lysostaphin (kSA2-E-Lyso-SH3b) or the staphylococcal phage K endolysin, LysK (kSA2-E-LysK-SH3b). These chimeric proteins successfully killed 16 mastitis *S. aureus* isolates. [87]. In addition, preliminary reports indicate that a recombinant endolysin Trx-SA1, derived from *S. aureus* bacteriophage IME-SA1, could constitute an effective treatment for dairy cow mastitis caused by *S. aureus* [89].

A series of studies were published on the use of phage endolysins to combat streptococci. Endolysins produced by streptococcal phages λSA2 and B30 were successfully applied as a treatment in a mouse model of bovine mastitis; both enzymes significantly reduced the intramammary pathogen concentration, in bacteria such as *S. dysgalactiae*, *S. uberis*, and *S. agalactiae*, and also ameliorated their effects on mammary glands [89]. Lysozymes were tested against a variety of animal models of mastitis; the results obtained indicated that both lysozymes and phage therapy represent an innovative alternative to antibiotics in the treatment of mastitis.

Moreover, several scientists have proposed the use of anti-virulence compounds that target a variety of virulence factors, either individually or in combination, which could represent a more effective therapy than current conventional treatments [91]. Virulence factor genes were studied in both *Staphylococcus aureus* and *S. uberis*, and these peptides, in both species, were considered to be putative targets for vaccines to prevent bovine mastitis. With this in mind, Collado et al. [92] and Perrig and colleagues [93], considered the possible use of additional *Staphylococcus* peptides, such as *S. uberis* adhesion molecule (SUAM), glyceraldehyde-3-phosphate dehydrogenase (GAPDH), fructose-biphosphate aldolase (FBA), elongation factor Ts (EFTs), mtuA, and an unspecified fibronectin-binding protein. In addition, several studies aimed to develop vaccines against a variety of *Streptococcus* group A proteins, including the M protein, pili components, adhesins, and the C5a protease [94,95,96,97,98,99].

In conclusion, two identified peptides, EATGNQNISPNLTISNAQLNLEDKNK and DLWC*NM*IIAAK, are highly specific for *S. dysgalactiae*, according to the GenBank database. These peptides could, therefore, be useful in strain bio-typing; furthermore, their use in combination with, yet to be described, additional proteins, could constitute a future strip-probe for the precise identification of *S. dysgalactiae*. In addition, specific peptides could be used to bind to aptamers, as they could be identified, directly from milk samples, through targeted mass spectrometry techniques. This technique could considerably reduce the time required for streptococci detection in foodstuffs, as the methods currently used are time-consuming. Moreover, the virulence factor peptides identified could be used as vaccines, and new enzybiotics, in the treatment of different mastitis-causing pathogenic bacteria; these peptides could even facilitate, in the near future, the diagnosis of hidden intracellular pathogenic streptococci, not currently detected by classical microbiological procedures.

## 4. Materials And Methods

### 4.1. Bacterial Strains

Table 2 summarizes the 14 *Streptococcus* spp. strains used in this study. Four reference strains (ST1, ST2, ST3, and ST14) were obtained from the Spanish culture collection, while ST4 and ST5 originated from the German culture collection. Strains ST6, ST7, ST8, ST9, ST10, and ST11 were isolated from the milk of cows suffering from mastitis by the LHICA (Laboratorio de Hygiene, Inspección y Control del Alimentos) at the Faculty of Veterinary Sciences, University of Santiago de Compostela, Spain. These bacterial strains were characterized, by the VITEK 2 system and 16S rRNA gene sequencing, and genetically identified as *Streptococcus* spp. [83]. The bacteria were grown in Brain Heart Infusion (BHI, Oxoid Ltd., Hampshire, UK), at 31 °C for 24 h. Bacterial cultures were then transferred to plate count agar (PCA, Oxoid Ltd., Hampshire, UK) and subjected to further incubation at 31 °C for 24 h.

### 4.2. Protein Extraction

Protein extraction was carried out as described previously [19,84]. In brief, an inoculation loop full of bacterial culture was harvested and the cells resuspended in 100 μL of a solution containing 50% acetonitrile (ACN; Merck, Darmstadt, Germany) and 1% aqueous trifluoroacetic acid (TFA; Acros Organics, NJ, US). After vortexing and centrifuging, the supernatant was treated with a solution of lysis buffer containing 60 mM Tris-HCl pH 7.5, 1% lauryl maltoside, 5 mM phenylmethanesulfonyl fluoride (PMSF) (Sigma, St. Louis, MO, US), and 1% dithiothreitol (DTT) (Sigma Chemical Co., US). The supernatant was then transferred to a fresh tube and the amount of protein was determined by the bicinchoninic acid method (Sigma Chemical Co., US). All experiments were performed in triplicates.

### 4.3. Peptide Sample Preparation

Protein extracts were subjected to in-solution tryptic digestion, as described previously [87]. Fractions containing 100 μg of protein were dried under vacuum in a SpeedVac (CentriVap, Labconco Co., US), resuspended in 25 μL of denaturation buffer, containing 8 M urea in 25 mM ammonium bicarbonate (pH 8.0), and sonicated for 5 min. This was followed by addition of DTT (final concentration of 10 mM) and incubation at 37 °C for 1 h. Alkylation was achieved by addition of iodoacetamide (Pierce, Thermo Fisher Scientific, San Jose, CA, US), to a final concentration of 50 mM, and the solution was incubated for 1 h at room temperature in the dark. The sample was then diluted with 4 volumes of 25 mM ammonium bicarbonate (pH 8.0), to reduce urea concentration. The final step involved protein digestion with trypsin (Promega, WI, US), to a final protease:protein ratio of 1:100, and incubation at 37 °C overnight.

### 4.4. Shotgun LC–ESI–MS/MS Analysis

The peptide digests obtained above were acidified with formic acid (FA) [100], desalted with a C18 MicroSpin™ column (The Nest Group, Southborough, MA, US), and analyzed by LC–ESI–MS/MS, using a Proxeon EASY-nLC II Nanoflow system (Thermo Fisher Scientific, San Jose, CA, US) coupled to an LTQ-Orbitrap XL mass spectrometer (Thermo Fisher Scientific) [19,20]. Peptide separation (2 μg) was performed on a reverse-phase (RP) column (EASY-Spray column, 50 cm × 75 μm ID, PepMap C18, 2 μm particles, 100 Å pore size, Thermo Fisher Scientific) equipped with a 10 mm precolumn (Accucore XL C18, Thermo Fisher Scientific). The column was eluted with a linear gradient from 5% to 35% solvent B (solvent A: 98% water, 2% ACN, 0.1% FA; solvent B: 98% ACN, 2% water, 0.1% FA), over 120 min at a flow rate of 300 nL/min. Electrospray ionization was carried out with a spray voltage of 1.95 kV at a capillary temperature of 230 °C. Peptides were analyzed in positive mode (1 μscan; 400 to 1600 amu), followed by 10 data-dependent collision-induced dissociation (CID) MS/MS scans (1 μscan), using an isolation width of 3 amu and a normalized collision energy of 35%. After the second fragmentation event, dynamic exclusion was set for 30 s, and ions with unassigned charge state were excluded from the MS/MS analysis.

### 4.5. LC–ESI–MS/MS Mass Spectrometry Data Processing

The amino acid sequences obtained for the peptides analyzed by LC–ESI–MS/MS were compared to the *Streptococcus* UniProt/TrEMBL database (containing 13,528 reviewed and 1,290,635 unreview protein sequence entries), using the tandem mass spectrometry data analysis program SEQUEST-HT (Proteome Discoverer 2.1 version, Thermo Fisher Scientific). MS/MS spectra were searched using fully tryptic cleavage constraints and up to two missed cleavage sites were allowed. Tolerance windows were set at 1.2 Da, for precursor ions, and 0.6 Da for MS/MS fragment ions. The variable modifications allowed in the tandem searches were: (M*) methionine oxidation (+ 15.99 Da), (C*) carbamidomethylation of Cys (+ 57.02 Da) and protein N-terminal acetylation (+ 42.0106 Da). The search results were validated by statistical analysis with the Percolator algorithm [101], keeping the peptide false discovery rate (FDR) less than 1%.

### 4.6. Determination of the Species Specificity of the Peptides Identified by LC–ESI–MS/MS

The peptides sequences obtained by LC–ESI–MS/MS were identified using the Basic Local Alignment Search Tool for proteins (BLASTp), which finds regions of homology with the protein sequences present in the NCBI database (https://blast.ncbi.nlm.nih.gov/Blast.cgi) [25]. To determine species specificity, the term *Streptococcaceae* was either included or excluded in the search, allowing the identification of peptides exclusively belonging to the *Streptococcus* spp.

## Figures and Tables

**Table 1:** *Streptococcus*-specific peptides, corresponding to virulence factors, identified in the *Streptococcus* spp. strains analyzed.

Function	Strain	Protein	Peptide	Identity by BLASTP
Toxins	ST1	Toxin RelE	LLATISM*IQEQGVLIAQRM*EWVKK	*Streptococcus suis*
	ST3	Antitoxin RelB	VFKENNLNTAQALNLFLKNVAETGQLNLK	*Streptococcus gallolyticus*
	ST12	Antitoxin YefM	NTYLSQKVLRGM*AK	*Streptococcus suis*
	ST2	Toxin YoeB	LIYM*M*DGDNVAFLSFKDHY	*Streptococcus mitis*
	ST11 and ST12	Pyrogenic exotoxin SpeK	NIYAPRYDEDEILDNR	*Streptococcus dysgalactiae* subsp. *dysgalactiae, Streptococcus dysgalactiae*
	ST9	Doc toxin	LYPTLFDKATILFVQLVKK	*Streptococcus sobrinus Streptococcus downei*
Antibiotic resistance	ST3	MarR family transcriptional regulator	M*DYQRINDYLTSIFNNVLVIEEM*SLRGSR	*Streptococcus* spp.
	ST4	MarR family transcriptional regulator	FNRFILAFEQLKK	*Streptococcus oralis*
	ST2	MarR family transcriptional regulator	EM*QQYVDLQGAYLALVKEEFAKAGLLPLK	*Streptococcus downei* MFe28
	ST9	MurM protein	QSLQRYLSEFRGFLDK	*Streptococcus equi*
	ST8	Beta-lactamase class A	FSITDVLVNSKKELVFQIDDK	*Streptococcus suis*
	ST6	Beta-lactamase class A	LVPDQPIQITGFYVNEEEVPIFKLKNGQFVIADK	*Streptococcus sanguinis*
	ST3	Cell wall-active antibiotics response protein	DTIHLERVILSNHDNVIILRK	*Streptococcus pseudopneumoniae, Streptococcus* sp. *HMSC061D10, Streptococcus* sp. *SK140*
	ST10	Streptomycin adenylyltransferase	M*RTETDM*FDVILQTAKVLQVDAVAM*SGSR	*Streptococcus cristatus*
	ST12	Penicillin binding protein	VQESAQNAGDTIGRAVK	*Streptococcus gallolyticus, Streptococcus macedonicus, Streptococcus pasteurianus*
	ST14	Glyoxalase/Bleomycin resistance protein	M*ITSLYPVLM*C*ENLEATANFFIENFQFR	*Streptococcus* sp. DD11
	ST1	M56 peptidase	FSHGQTAHETIVNAKDGKLVK	*Streptococcus sanguinis*
Resistance to toxic substances	ST4	TelA protein	DSLQEFYFDSKSIEQKM*DGM*AAAVVK	*Streptococcus iniae*
	ST1	MerR family transcriptional regulator	LEDHLLDLKAK	*Streptococcus agalactiae, Streptococcus halotolerans, Streptococcus thoraltensis, Streptococcus acidominimus*
Colonization and immune evasion	ST1	N-acetylmuramoyl-L-alanine amidase	M*KKVILASTVALSILGFTQATVQAQENNAESVR	*Streptococcus mitis*
	ST7	N-acetylmuramoyl-L-alanine amidase	LVEIAFIDNNSDM*ATYEANK	*Streptococcus dysgalactiae, Streptococcus urinalis, Streptococcus porcinus, Streptococcus agalactiae, Streptococcus pluranimalium, Streptococcus suis*
	ST8	Lysin	AGAIFVKREASHDYGHTGVVIK	*Streptococcus phocae*
	ST10	Lysozyme	LIIFLLVFLFAFQTYR	*Streptococcus henryi*
	ST4	Lysozyme M1 (1,4-beta-N-acetylmuramidase)	LNPM*IVVVFFLSFFALIFITGVTGNTVNK	*Streptococcus suis*
	ST3	CLpX ATPases	EENDVDLQKSNILM*IGPTGSGKTFLAQTLAR	*Streptococcus vestibularis*
	ST5	CLpX ATPases	SIIEETM*LDVM*FEVPSQENVKLIRITK	*Streptococcus pneumoniae*
	ST5	CLp ATPases	WIGDAQKRTK	*Streptococcus agalactiae, Streptococcus canis, Streptococcus equi, Streptococcus castoreus, Streptococcus dysgalactiae*
	ST9	CLp ATPases	RTIQDHIEDAITDYYLEHPK	*Streptococcus cristatus, Streptococcus gordonii*
	ST8	CLp ATPases	ENLLQIVELM*LADVNKRLSSNNIHLDVTDK	*Streptococcus pneumoniae, Streptococcus mitis*
	ST8	CLpC ATPases	EDVVKLIGNRATR	*Streptococcus sinensis, Streptococcus anginosus*
	ST14	CLpX ATPases	NNPVLVGDAGVGKTVLALGLAQR	*Streptococcus suis, Streptococcus pneumoniae*
	ST3	CLp ATPases	IM*VQPLIAHLAEKNISLK	*Streptococcus macacae*
	ST7	CLp ATPases	ETIKAIHDLRKPK	*Streptococcus castoreus, Streptococcus ictaluri*
	ST6	Neuraminidase A	SLVLPKLPGQVSLIGSNKQGVVDLNNK	*Streptococcus* sp. HMSC074B11, *Streptococcus pseudopneumoniae, Streptococcus* sp. HPH0090, *Streptococcus* sp. *oral taxon 431, Streptococcus mitis, Streptococcus* sp. *UMB0029, Streptococcus* sp. LQJ-218, *Streptococcus infantis*
	ST6	Sialidase B	NAPYLGPGRGIIESSTGRILIPSYTGK	*Streptococcus pneumoniae, Streptococcus mitis, Streptococcus pseudopneumoniae, Streptococcus infantis*
	ST13	Sialidase A	VPLVTSGDYSGSPINM*DM*ALVQDTSSKTK	*Streptococcus agalactiae*
	ST14	Sialidase A	VPTLQLANGKTARFM*TQYDTK	*Streptococcus pneumoniae, Streptococcus oralis*
	ST3	Sialidase A	EDVETNTSNGQRVDLSSELDKLK	*Streptococcus pneumoniae*
	ST10	Choline binding protein (Cbp)	TGWVKDKGTWYYLDK	*Streptococcus pneumoniae*
	ST2	Choline binding protein (Cbp)	EGSTWYYLKGSGAM*ATGWATANGQWSYFEK	*Streptococcus mitis*
	ST7	PspA	TEQVLLTEAVQQVQR	*Streptococcus gordonii, Streptococcus cristatus*
	ST4	PspA	DLDAADKALEAAQAELKAR	*Streptococcus mitis*
	ST4	Ig A1 protease	GTESEAAKPAPKEAGTTAGNEVK	*Streptococcus pneumoniae*
	ST2	Ig A1 protease	NNDKYYAIYNLK	*Streptococcus* sp. 596553, *Streptococcus pneumoniae*
	ST2	Ig A1 protease	KKVM*GLLLIGSM*GQSLLLSIDAAALQNIELR	*Streptococcus* spp.
	ST13	Sortase A	AKVGM*TIYLTDKSM*IYTYK	*Streptococcus gallolyticus, Streptococcus macedonicus, Streptococcus pasteurianus, Streptococcus henryi*
	ST2	Sortase B	NFLIGQQSNHYQVSKVSKK	*Streptococcus macedonicus, Streptococcus gallolyticus, Streptococcus pasteurianus, Streptococcus lutetiensis,*
	ST4	Sortase A	YYYEAAFLIIVPENTAFYK	*Streptococcus azizi, Streptococcus acidominimus*
	ST6 and ST13	C5A peptidase	EDISGEEASAPQTSPQESPVEPEEVTRGR	*Streptococcus suis*
	ST2	C5A peptidase	YPDKSPAEISELVKALIM*STAKPHINK	*Streptococcus anginosus*
	ST13	M protein	LM*EERARHVDLIDNIR	*Streptococcus pyogenes*
	ST1	M Protein	SVAVAVAVLGAAFANQTEVK	*Streptococcus pyogenes*
	ST1	M Protein	AEAVSRSNSEQNNLEKR	*Streptococcus pyogenes*
	ST14	M Protein	IVAVALTVVGAGFANQTEVK	*Streptococcus pyogenes*
	ST11	M Protein	YVEKSYHLLSDFIDQISSTYNFKIDNK	*Streptococcus cristatus*
	ST9	Mga protein	KVLLTFFLDKR	*Streptococcus pseudoporcinus*
	ST5	O-acetylase OafA	IVPPLVM*M*ILLIIPFTFLVR	*Streptococcus henryi*
	ST10	Superoxide dismutase	FGSGWAWLVVNPDGKLEVM*STANQDTPISEGK	*Streptococcus anginosus, Streptococcus anginosus* subsp. *anginosus, Streptococcus constellatus* subsp. *constellatus, Streptococcus* sp. 8400103
	ST14	Superoxide dismutase	FGSGWAWLVVNKDGKLEVTSTANQDTPLSEGK	*Streptococcus infantarius, Streptococcus equinus*
	ST6	Peptidoglycane-N-acetylglucosamine deacetylase	DAELYQTYFAQK	*Streptococcus oralis*
	ST6	CpsB	KGM*FETPEEKIAENFLQIR	*Streptococcus pneumoniae*
	ST1	CpsC	EIILSQDVLEKVATDLKLELPPK	*Streptococcus* sp. 1643, *Streptococcus oralis*
	ST5	CpsC	EIIISQDVLEEVVSDLKLDLTPK	*Streptococcus pneumoniae*
	ST13	CapD protein	KLTDYVIDLVEILNK	*Streptococcus pneumoniae, mitis, Streptococcus pseudopneumoniae, Streptococcus oralis, Streptococcus australis, Streptococcus* sp. M334
	ST3	Accessory pilus subunit	NNVKTYLLKIK	*Streptococcus suis*
	ST8	Pilin protein FimC	SRFGDAADKAASLSAK	*Streptococcus sanguinis*
	ST12	Agglutinin receptor	TVETIQSTNEQAVADYLTKKTK	*Streptococcus suis*, *Streptococcus agalactiae*
	ST3	Agglutinin receptor	VESAVSLAKEAGLTVK	*Streptococcus mitis*
	ST7	Agglutinin receptor	TIDPSVHQYGQQELDALVK	*Streptococcus oralis, Streptococcus* sp. CM6, *Streptococcus* sp. SR1
	ST9	Agglutinin receptor	TTSLM*FEDYLPAGYLFDLEKTLAENGDYEVTFDASK	*Streptococcus canis* FSL Z3-227
	ST8	adhesin P1/ Cell surface antigen I/II	ADYEAKLAKYQADLAK	*Streptococcus mutans, Streptococcus intermedius, Streptococcus anginosus*
	ST7	CppA protein	NLFQGRENFIPK	*Streptococcus anginosus*
	ST9	Transposase TcpC	TLEQFLDGYVSRYFTYDSQAGSSDENISK	*Streptococcus pneumoniae, Streptococcus oralis, Streptococcus* sp. HMSC056C01, *Streptococcus* sp. SK140, *Streptococcus infantis* SK1302
	ST5	LytR family transcriptional regulator	AHTVQIITEEASFNM*VQNLSNLENQYGETLM*R	*Streptococcus oralis*
	ST2	Asp23 protein	SGLSGGFSAVQEKVGEGVEAVKDAASSNENTR	*Streptococcus cristatus*
	ST12	Asp23protein	KM*TDLDVIEVNVKVVDIK	*Streptococcus phocae, Streptococcus canis, Streptococcus ictaluri, Streptococcus pyogenes, Streptococcus dysgalactiae, Streptococcus dysgalactiae* subsp. *equisimilis, Streptococcus dysgalactiae* subsp. *dysgalactiae, Streptococcus dysgalactiae* subsp. *equisimilis* SK1249
	ST14	Asp23 protein	ATEDGSIAVDVYTVLSYGTKISEVSKNIQER	*Streptococcus infantis, Streptococcus oralis, Streptococcus mitis*
	ST2	Type VII secretion protein EsaA	NSDVSTALSNIWFEAIDSNLKK	*Streptococcus oralis*
	ST2	Type VII secretion protein EssB	LRLALNLLDLEQALSLPVTFFLHPENLFITK	*Streptococcus pantholopis*
	ST8	Type VII secretion protein EssB	LEFVREDNQISVQISSSGYRR	*Streptococcus* sp., *Streptococcus mitis*
	ST14	Virulence factor	VFGQTDETTIPLLANALADSM*NQSELETLPR	*Streptococcus macedonicus, Streptococcus equinus*
	ST3	Virulence-associated protein E	M*KATVDNYVLVLRNDPYISESLK	*Streptococcus pasteurianus*
	ST9	Equibactin	LYEISLKVADC*LGKNGVK	*Streptococcus equi*
Antimicrobial production	ST3	Bacteriocin	WTSKSSKAYAYAGQTSYAFIK	*Streptococcus salivarius*
	ST2	Bacteriocin	M*SQKIGIM*M*NIK	*Streptococcus intermedius*
	ST14	Bacteriocin-associated integral membrane protein	AIAVGFSLAGVLAILM*QK	*Streptococcus pneumoniae*
	ST4	LanT protein	QNVDKLHFTRFDK	*Streptococcus pneumoniae*
	ST12	LanM protein	RAATKFM*INTDC*PSK	*Streptococcus pneumoniae*
ABC Transporters	ST2	Metal ABC transporter	DGADYISVM*QDNLKALEK	*Streptococcus varani*
	ST6	Metal ABC transporter	VPSAYIWEINTEEEGTPDQISSLIEK	*Streptococcus pyogenes, Streptococcus equi* subsp. *zooepidemicus* Sz105, *Streptococcus canis, Streptococcus castoreus, Streptococcus porcinus, Streptococcus ictaluri, Streptococcus equi*
	ST10	Copper ABC transporter	SM*PDAIYLFTLLKVAC*M*GLTSFYSLR	*Streptococcus infantarius, Streptococcus lutetiensis, Streptococcus equinus, Streptococcus* sp. CNU 77-61, *Streptococcus* sp. KCJ4932
	ST1	Copper ABC transporter	NNLTLYENQYSLPIAFASQSIYNNVK	*Streptococcus mitis*
	ST10	Zinc ABC transporter	AVIARM*FASDPNIFVLDEPTTGM*DAGSK	*Streptococcus spp.*
	ST3	Zinc ABC transporter	TIYKNFM*EIGTAILM*STGLAISLIVM*SKGK	*Streptococcus cristatus, Streptococcus* sp. HMSC062B01, *Streptococcus gordonii,*
	ST2	Cobalt or another cation ABC transporter	DGKLREVFQIPSYEM*TQVASK	*Streptococcus pneumoniae*
	ST3	Cobalt ABC transporter	LSSDPVEVTQYYIEKGGPNV	*Streptococcus salivarius*
	ST2	Cobalt ABC transporter (CbiM)	IISKDPNSKTM*LALSGAFIFILSSLK	*Streptococcus australis, Streptococcus parasanguinis*
	ST4	FeoABC transporter (FeoB)	LM*DM*GLTHHTKIYLRK	*Streptococcus gallolyticus*
	ST9	FeoABC transporter (FeoB)	EATGNQNISPNLTISNAQLNLEDKNK	*Streptococcus dysgalactiae*
	ST1	Bacitracin ABC transporter (BceAB)	TVLGFGC*FVVQLVVIILVAYANGYVM*K	*Streptococcus* sp. HSISM1, *Streptococcus parasanguinis*
	ST14	Bacitracin ABC transporter (BceAB)	QNIIALIQENGIKKSVLAK	*Streptococcus* sp. SK643, *Streptococcus pseudopneumoniae*
	ST2	Bacitracin ABC transporter	SVEYPEKIATLLVNAGYPPK	*Streptococcus sanguinis*
	ST9 and ST12	Bacteriocin ABC transporter	VNKGEFIAIM*GESGSGK	*Streptococcus phocae*
	ST9	Bacteriocin ABC transporter	M*IVNFYTPNHGQITLGDYDLK	*Streptococcus gallolyticus*
	ST4	Bacteriocin ABC transporter	KTVEDLSM*M*KGDM*TFK	*Streptococcus oralis, Streptococcus* sp. NPS 308, *Streptococcus sp. oral taxon 071* str. 73H25AP, *Streptococcus mitis, Streptococcus* sp. VT 162, *Streptococcus australis, Streptococcus pseudopneumoniae, Streptococcus halitosis, Streptococcus* spp.
	ST9	Lantibiotic Mutacin ABC transporter protein (MutE)	LM*VPILNILPNGLPAGTDAVVAPK	*Streptococcus sobrinus*
	ST4	Lantibiotic ABC transporter	STIM*KIIFGLENADSGAIVFNGGKNAGK	*Streptococcus mitis*
	ST14	Amino acid ABC transporter	M*VDGKNQVVGADIGM*AQAIADELGVK	*Streptococcus oralis*
	ST3	Amino acid ABC transporter	NLTDKSQM*NIGIFFAIIALVVIWFLM*KK	*Streptococcus parasanguinis*
	ST13	Amino acid ABC transporter	TGVPLLTPSGTQDDLTVDAK	*Streptococcus* sp. 449_SSPC, *Streptococcus salivarius*
	ST14	Amino acid ABC transporter	VIFM*DKGIIAEEGKPEDLFTNPKEER	*Streptococcus sp. oral taxon 058, Streptococcus oralis*
	ST13	Amino acid ABC transporter	IVLPQAFRIALPNLTTALLNLM*R	*Streptococcus* sp. AS14, *Streptococcus sanguinis, Streptococcus cristatus, Streptococcus* sp. CCH8-C6
	ST9, ST13 and ST14	Amino acid ABC transporter	NLLLAPVKVQKR	*Streptococcus* sp. 45, *Streptococcus infantarius, Streptococcus* sp. KCJ4932 *Streptococcus infantarius subsp. infantarius* CJ18, *Streptococcus lutetiensis* 033, *Streptococcus infantarius, Streptococcus equinus*
	ST10	Glutamine ABC transporter	DASLAPM*FVAGAIYLIM*IGLVTLISKQVEK	*Streptococcus* sp. DD13
	ST13	Glutamine ABC transporter	KDEVIKEAENLLER	*Streptococcus sanguinis*
	ST14	Glycine/betaine ABC transporter	YDLQVLEDDKQLFPPYQGAPLM*KEDLLK	*Streptococcus oralis, Streptococcus mitis*
	ST2	Glycine/betaine ABC transporter	QEITLAYVEWDSEVASTNVLAEVLKTK	*Streptococcus infantarius*
	ST4	Glycine/betaine ABC transporter	AKLRTIVAAFAVM*VLGLGASYAPSM*IPSK	*Streptococcus infantis*
	ST14	Oligopeptide ABC transporter	KNVQM*IFQDPQASLNAR	*Streptococcus infantarius, Streptococcus lutetiensis*
	ST1	Multidrug ABC transporter	QLQQYIYESLLTTSVK	*Streptococcus suis*
	ST4	Multidrug ABC transporter	SGSKALKQLQQYIYESLLTTSVK	*Streptococcus suis*
	ST10	Multidrug ABC transporter	LESKEIDENSIVSK	*Streptococcus pneumoniae, Streptococcus salivarius, Streptococcus* sp. HMSC068F04, *Streptococcus* sp. FDAARGOS_192, *Streptococcus* sp. SR4, * thermophilus, Streptococcus* sp. C150, *Streptococcus* sp. HMSC064H09, *Streptococcus* sp. HMSC064H03, *Streptococcus* sp. HSISS2
	ST2	Multidrug ABC transporter	AQGTLADLQATFGDASASLNDIYLALTKEV	*Streptococcus phocae*
	ST1	Multidrug ABC transporter	YLLNLDEKQINIAPHLTINHLK	*Streptococcus*
	ST1	Multidrug ABC transporter	M*PTAFYLFFSSM*YQDTPGGPANFM*R	*Streptococcus pneumoniae*
	ST5	Multidrug ABC transporter	TTLIM*VSQRTNSLAK	*Streptococcus sp. ‘caviae’*
	ST7	Multidrug ABC transporter	FPNAFYLSM*SILLVQAVLNM*R	*Streptococcus pantholopis*
	ST3	Multidrug ABC transporter	SGVVLSLLGAM*ISFILYLVFLKANIK	*Streptococcus* sp. HMSC066E07, *Streptococcus anginosus*
		Multidrug ABC transporter	IAYLPQEGALFHDTVLYNLTIGREVPEDR	Streptococcus suis
	ST14	Macrolide ABC transporter (MacB)	STLM*NIIGM*LDRPTSGEYYLEGEEVAKLSEK	*Streptococcus anginosus, Streptococcus* sp. KCOM 2412, *Streptococcus* sp. HMSC057E02
Other Transporters	ST2	Manganese transport protein MntH	YLLLSVVLISSLIAM*QLQQM*AGKLGIVTQK	*Streptococcus equinus, Streptococcus* sp. KCJ4950
	ST6	HlyC/CorC family transporter	TAPVIIFLGKIVSPFVWLLSASTNLLSQM*TPM*K	*Streptococcus cristatus, Streptococcus* sp. marseille-P644, *Streptococcus sp.* marseille-P7375
	ST9	Multidrug transporter MatE	AM*LIM*SLGAGINIVLDPVLM*IM*FK	*Streptococcus intermedius, Streptococcus* sp. AS20
	ST14	MFS Lantibiotic transporter	DLWC*NM*IIAAK	*Streptococcus dysgalactiae*

(M* methionine oxidation; C* carbamidomethylation of Cys).

**Table 2:** *Streptococcus* spp. strains used in this study. Total peptides represents the number of peptides identified by LC–ESI–MS/MS and those analyzed by BLASTp. ATCC—American Type Culture Collection; CECT—Spanish Type Culture Collection; DSM—German Type Culture Collection.

Sample	Species	Strain	Source	NCBI Accession No. of 16S RNA Gene	Total Peptides
**ST1**	*Streptococcus uberis*	CECT 994	unknown	JN630842.1	282
**ST2**	*Streptococcus agalactiae*	CECT 183	Milk	KC510212.1	409
**ST3**	*Streptococcus dysgalactiae subsp. equisimilis*	CECT 926	unknown		259
**ST4**	*Streptococcus parauberis*	DSM 6631	Mastitis sample milk	NR_043001.1	221
**ST5**	*Streptococcus parauberis*	DSM 6632	Raw milk	JN630844.1	134
**ST6**	*Streptococcus agalactiae*	USC1-LHICA	Mastitis sample milk	KP001323.1	140
**ST7**	*Streptococcus agalactiae*	USC3-LHICA	Mastitis sample milk	KC510215.1	169
**ST8**	*Streptococcus uberis*	USC5-LHICA	Mastitis sample milk	KC510216.1	131
**ST9**	*Streptococcus dysgalactiae subsp. dysgalactiae*	USC13-LHICA	Mastitis sample milk	KC510218.1	249
**ST10**	*Streptococcus canis*	USC52-LHICA	Mastitis sample milk	KC510222.1	247
**ST11**	*Streptococcus uberis*	USC69-LHICA	Mastitis sample milk	KC510224.1	44
**ST12**	*Streptococcus gallolyticus subsp. gallolyticus*	USC83-LHICA	Mastitis sample milk	KC510227.1	154
**ST13**	*Streptococcus gallolyticus subsp. gallolyticus*	USC84	Mastitis sample milk	KC51022.8	148
**ST14**	*Streptococcus dysgalactiae subsp. dysgalactiae*	CECT 758	Mastitis sample milk	KC51021.3	238

## Supplementary Materials

The following are available online at https://www.mdpi.com/2079-6382/9/6/302/s1, Table S1, Excel data sheet in Supplementary Data 1: Complete lists of un-redundant peptides identified for the complete dataset and for each *Streptococcus* spp. Table S2, Supplementary Data 2: Peptides, identified in the Streptococcus spp. strains analyzed, which represent the virulence factors.
