# Supplementary material for: Proteomic Characterization of Antibiotic Resistance, and Production of Antimicrobial and Virulence Factors in Streptococcus Species Associated with Bovine Mastitis. Could Enzybiotics Represent Novel Therapeutic Agents Against These Pathogens?
Source: Antibiotics (Basel). 2020 Jun 4;9(6):302. doi: 10.3390/antibiotics9060302 (PMC7344566; doi:10.3390/antibiotics9060302)
Supplement: Supplementary file 1 [file antibiotics-09-00302-s001.zip › Supplementary Data 2.docx]

Supplementary Data 2

Table S2. Peptides, identified in the *Streptococcus* spp. strains analyzed, that represent virulence factors.

| **Function** | **Strain** | **Protein** | **Peptide** | **100% sequence identity by NCBI** |
| --- | --- | --- | --- | --- |
| Toxins | ST1 | Toxin RelE | LLATISM*IQEQGVLIAQRM*EWVKK | *Streptococcus suis* |
|  | ST3 | Antitoxin RelB | VFKENNLNTAQALNLFLKNVAETGQLNLK | *Streptococcus gallolyticus* |
|  | ST12 | Antitoxin YefM | NTYLSQKVLRGM*AK | *Streptococcus suis* |
|  | ST2 | Toxin YoeB | LIYM*M*DGDNVAFLSFKDHY | *Streptococcus mitis* |
|  | ST11 and ST12 | Pyrogenic exotoxin SpeK | NIYAPRYDEDEILDNR | *Streptococcus dysgalactiae* subsp. *dysgalactiae,*  *Streptococcus dysgalactiae* |
|  | ST3 | Beta-class phenol-soluble modulin | LGTSIVDIVESGVSVLGK | *Staphylococcus epidermidis* |
|  | ST9 | Doc toxin | LYPTLFDKATILFVQLVKK | *Streptococcus sobrinus Streptococcus downei* |
| Antibiotic resistance | ST3 | MarR family transcriptional regulator | M*DYQRINDYLTSIFNNVLVIEEM*SLRGSR | *Streptococcus* spp. |
|  | ST4 | MarR family transcriptional regulator | FNRFILAFEQLKK | *Streptococcus oralis* |
|  | ST2 | MarR family transcriptional regulator | EM*QQYVDLQGAYLALVKEEFAKAGLLPLK | *Streptococcus downei* MFe28 |
|  | ST9 | MurM protein | QSLQRYLSEFRGFLDK | *Streptococcus equi* |
|  | ST8 | Beta-lactamase class A | FSITDVLVNSKKELVFQIDDK | *Streptococcus suis* |
|  | ST6 | Beta-lactamase class A | LVPDQPIQITGFYVNEEEVPIFKLKNGQFVIADK | *Streptococcus sanguinis* |
|  | ST3 | TipAS antibiotic-recognition domain | EVASDEVQGATKRLM*QK | *Bacillus cereus, Bacillus thuringiensis, Bacillus anthracis, Bacillus tropicus, Streptococcus pneumoniae* |
|  | ST3 | Cell wall-active antibiotics response protein | DTIHLERVILSNHDNVIILRK | *Streptococcus pseudopneumoniae, Streptococcus* sp. HMSC061D10*, Streptococcus* sp. SK140 |
|  | ST10 | Streptomycin adenylyltransferase | M*RTETDM*FDVILQTAKVLQVDAVAM*SGSR | *Streptococcus cristatus* |
|  | ST1 | Penicillin binding protein | ASKEEILTFYINKVYM*ANGYYGM*R | *Floricoccus penangensis* |
|  | ST12 | Penicillin binding protein | VQESAQNAGDTIGRAVK | *Streptococcus gallolyticus, Streptococcus macedonicus, Streptococcus pasteurianus* |
|  | ST14 | Glyoxalase/Bleomycin resistance protein | M*ITSLYPVLM*C*ENLEATANFFIENFQFR | *Streptococcus* sp. *DD11* |
|  | ST1 | M56 peptidase | FSHGQTAHETIVNAKDGKLVK | *Streptococcus sanguinis* |
| Other resistances | ST4 | TelA protein | DSLQEFYFDSKSIEQKM*DGM*AAAVVK | *Streptococcus iniae* |
|  | ST9 | MerA mercuric reductase | LLKEYDPEISEAITK | *Streptococcus mitis* SK579, *Streptococcus mitis, Streptococcus pneumoniae, Streptococcus mitis* SK1073, *Streptococcus oralis, Weissella thailandensis, Enterococcus faecium, Aerococcus* sp. *1KP-2016, Dialister micraerophilus, Rothia* sp. HMSC065G12*, Bacillus cereus,Leuconostocaceae bacterium, Streptococcus gordonii, Lactobacillus parafarraginis, Solibacillus isronensis, Bacillus megaterium, Pantoea agglomerans.* |
|  | ST1 | MerR family transcriptional regulator | LEDHLLDLKAK | *Streptococcus agalactiae, Streptococcus halotolerans, Streptococcus thoraltensis, Streptococcus acidominimus* |
| Colonization and immune evasion | ST1 | N-acetylmuramoyl-L-alanine amidase | M*KKVILASTVALSILGFTQATVQAQENNAESVR | *Streptococcus mitis* |
|  | ST8 | N-acetylmuramoyl-L-alanine amidase | LIKPQPKPQPQPQPKPQTKPVSK | *Peptostreptococcus anaerobius* |
|  | ST10 | N-acetylmuramoyl-L-alanine amidase | VLKHIEDESLIK | *Peptostreptococcus anaerobius* |
|  | ST7 | N-acetylmuramoyl-L-alanine amidase | LVEIAFIDNNSDM*ATYEANK | *Streptococcus dysgalactiae, Streptococcus urinalis, Streptococcus porcinus, Streptococcus agalactiae, Streptococcus pluranimalium, Streptococcus suis* |
|  | ST14 | N-acetylmuramoyl-L-alanine amidase | SM*IKTAILSLIAIFVIVPTASADNSVSRIDGGSR | *Bacillus altitudinis, Bacillus* spp. |
|  | ST2 | LysM domain | TLNSLKSDTIYPNQVLK | *Bacillus* spp. |
|  | ST5 | LysM domain | VEEPATPAPKAEEPATPAPK | *Streptococcus mitis, Gemella haemolysans, Streptococcus pseudopneumoniae, Streptococcus oralis* |
|  | ST6 | Bifunctional autolysin | KGSIIGLIGLLIILVAAGFIFFSM*ISDQIFFKK | *Staphylococcus hominis, Mycobacteroides abscessus sub*sp. *Abscessus, Bacilli,* |
|  | ST8 | Lysin | AGAIFVKREASHDYGHTGVVIK | *Streptococcus phocae* |
|  | ST10 | Lysozyme | LIIFLLVFLFAFQTYR | *Streptococcus henryi* |
|  | ST4 | Lysozyme M1 (1,4-beta-N-acetylmuramidase) | LNPM*IVVVFFLSFFALIFITGVTGNTVNK | *Streptococcus suis* |
|  | ST3 | CLpX ATPases | EENDVDLQKSNILM*IGPTGSGKTFLAQTLAR | *Streptococcus vestibularis* |
|  | ST5 | CLpX ATPases | SIIEETM*LDVM*FEVPSQENVKLIRITK | *Streptococcus pneumoniae* |
|  | ST5 | CLp ATPases | WIGDAQKRTK | *Streptococcus agalactiae, Streptococcus canis, Streptococcus equi,*  *Streptococcus castoreus, Streptococcus dysgalactiae* |
|  | ST9 | CLp ATPases | RTIQDHIEDAITDYYLEHPK | *Streptococcus cristatus, Streptococcus gordonii* |
|  | ST8 | CLp ATPases | ENLLQIVELM*LADVNKRLSSNNIHLDVTDK | *Streptococcus pneumoniae, Streptococcus mitis* |
|  | ST8 | CLpC ATPases | EDVVKLIGNRATR | *Streptococcus sinensis, Streptococcus anginosus* |
|  | ST14 | CLpX ATPases | NNPVLVGDAGVGKTVLALGLAQR | *Streptococcus suis, Streptococcus pneumoniae* |
|  | ST3 | CLp ATPases | IM*VQPLIAHLAEKNISLK | *Streptococcus macacae* |
|  | ST9 | CLpX ATPases | SIIEEIM*M*DVM*FDVPSDESIEKVIITK | *Enterococcus gallinarum, Enterococcus faecalis, Listeria monocytogenes, Bacilli* |
|  | ST7 | CLp ATPases | ETIKAIHDLRKPK | *Streptococcus castoreus, Streptococcus ictaluri* |
|  | ST3 | CLpC ATPases | IDEIIVFHSLEKKHLTEIVSLM*SDQLTK | *Pseudomonas* sp. *GW456-E7 Bacillus vallismortis, Bacillus subtilis, Bacillus intestinalis Bacillus tequilensis* |
|  | ST6 | Neuraminidase A | SLVLPKLPGQVSLIGSNKQGVVDLNNK | *Streptococcus* sp. HMSC074B11, *Streptococcus pseudopneumoniae, Streptococcus* sp. HPH0090, *Streptococcus* sp*.* oral taxon 431, *Streptococcus mitis, Streptococcus* sp. UMB0029, *Streptococcus* sp. LQJ-218, *Streptococcus infantis* |
|  | ST6 | Sialidase B | NAPYLGPGRGIIESSTGRILIPSYTGK | *Streptococcus pneumoniae, Streptococcus mitis, Streptococcus pseudopneumoniae,*  *Streptococcus infantis* |
|  | ST13 | Sialidase A | VPLVTSGDYSGSPINM*DM*ALVQDTSSKTK | *Streptococcus agalactiae* |
|  | ST14 | Sialidase A | VPTLQLANGKTARFM*TQYDTK | *Streptococcus pneumoniae, Streptococcus oralis* |
|  | ST3 | Sialidase A | EDVETNTSNGQRVDLSSELDKLK | *Streptococcus pneumoniae* |
|  | ST10 | Choline binding protein (Cbp) | TGWVKDKGTWYYLDK | *Streptococcus pneumoniae* |
|  | ST2 | Choline binding protein (Cbp) | EGSTWYYLKGSGAM*ATGWATANGQWSYFEK | *Streptococcus mitis* |
|  | ST7 | PspA | TEQVLLTEAVQQVQR | *Streptococcus gordonii, Streptococcus cristatus* |
|  | ST4 | PspA | DLDAADKALEAAQAELKAR | *Streptococcusmitis* |
|  | ST4 | Ig A1 protease | GTESEAAKPAPKEAGTTAGNEVK | *Streptococcus pneumoniae* |
|  | ST2 | Ig A1 protease | NNDKYYAIYNLK | *Streptococcus* sp*. 596553, Streptococcus pneumoniae* |
|  | ST2 | Ig A1 protease | KKVM*GLLLIGSM*GQSLLLSIDAAALQNIELR | *Streptococcus* spp. |
|  | ST13 | Sortase A | AKVGM*TIYLTDKSM*IYTYK | *Streptococcus gallolyticus, Streptococcus macedonicus, Streptococcus pasteurianus, Streptococcus henryi,* |
|  | ST14 | Sortase C | M*IGAGAIIVGAVLFALYR | *Bacillus cereus* |
|  | ST2 | Sortase B | NFLIGQQSNHYQVSKVSKK | *Streptococcus macedonicus, Streptococcus gallolyticus, Streptococcus pasteurianus, Streptococcus lutetiensis,* |
|  | ST4 | Sortase A | YYYEAAFLIIVPENTAFYK | *Streptococcus azizi,*  *Streptococcus acidominimus* |
|  | ST6 and ST13 | C5A peptidase | EDISGEEASAPQTSPQESPVEPEEVTRGR | *Streptococcus suis* |
|  | ST2 | C5A peptidase | YPDKSPAEISELVKALIM*STAKPHINK | *Streptococcus anginosus* |
|  | ST13 | M protein | LM*EERARHVDLIDNIR | *Streptococcus pyogenes* |
|  | ST1 | M Protein | SVAVAVAVLGAAFANQTEVK | *Streptococcus pyogenes* |
|  | ST1 | M Protein | AEAVSRSNSEQNNLEKR | *Streptococcus pyogenes* |
|  | ST14 | M Protein | IVAVALTVVGAGFANQTEVK | *Streptococcus pyogenes* |
|  | ST11 | M Protein | YVEKSYHLLSDFIDQISSTYNFKIDNK | *Streptococcus cristatus* |
|  | ST9 | Mga protein | KVLLTFFLDKR | *Streptococcus pseudoporcinus* |
|  | ST14 | Mga protein | VLEKIAPYFDM*PHDKIVK | *Weissella confusa,*  *Streptococcus pneumoniae* |
|  | ST7 | Mga protein | KHHLALSERLVLTGDEISVR | *Weissella confusa,*  *Streptococcus pneumoniae* |
|  | ST5 | O-acetylase OafA | IVPPLVM*M*ILLIIPFTFLVR | *Streptococcus henryi* |
|  | ST10 | Superoxide dismutase | FGSGWAWLVVNPDGKLEVM*STANQDTPISEGK | *Streptococcus anginosus, Streptococcus anginosus sub*sp. *anginosus, Streptococcus constellatus* subsp. *constellatus, Streptococcus* sp. 8400103 |
|  | ST14 | Superoxide dismutase | FGSGWAWLVVNKDGKLEVTSTANQDTPLSEGK | *Streptococcus infantarius, Streptococcus equinus* |
|  | ST6 | Peptidoglycane-N-acetylglucosamine deacetylase | DAELYQTYFAQK | *Streptococcus oralis* |
|  | ST4 | Type II secretion system protein F | QFLLPQLM*ENDANSYSK | *Enterococcus faecalis* |
|  | ST6 | CpsB | KGM*FETPEEKIAENFLQIR | *Streptococcus pneumoniae* |
|  | ST1 | CpsC | EIILSQDVLEKVATDLKLELPPK 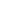 | *Streptococcus* sp. 1643, *Streptococcus oralis* |
|  | ST5 | CpsC | EIIISQDVLEEVVSDLKLDLTPK | *Streptococcus pneumoniae* |
|  | ST13 | CapD protein | KLTDYVIDLVEILNK 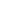 | *Streptococcus pneumoniae, mitis, pseudopneumoniae, oralis, australis, Streptococcus* sp. M334 |
|  | ST3 | Accessory pilus subunit | NNVKTYLLKIK | *Streptococcus suis* |
|  | ST13 | Flp pilus assembly protein CpaB | KEELPDSAILNLK | *Bacillus* spp. |
|  | ST8 | Pilin protein FimC | SRFGDAADKAASLSAK | *Streptococcus sanguinis* |
|  | ST12 | Agglutinin receptor | TVETIQSTNEQAVADYLTKKTK | *Streptococcus suis,*  *Streptococcus agalactiae* |
|  | ST3 | Agglutinin receptor | VESAVSLAKEAGLTVK | *Streptococcus mitis* |
|  | ST7 | Agglutinin receptor | TIDPSVHQYGQQELDALVK | *Streptococcus oralis, Streptococcus* sp. CM6, *Streptococcus* sp. SR1 |
|  | ST9 | Agglutinin receptor | TTSLM*FEDYLPAGYLFDLEKTLAENGDYEVTFDASK | *Streptococcus canis* FSL Z3-227 |
|  | ST2 | Bacillolysin | NPDWEIGEDIYTPGK | *Bacillus* spp. |
|  | ST1 | Collagen adhesion protein | VTVVAGQVAKVNFNNVLK | *Streptococcus suis, Eubacterium* sp. *marseille-*P5640*, Bariatricus massiliensis, Clostridium innocuum, Faecalicatena orotica, Lachnospiraceae bacterium, Pseudoflavonifractor* sp. *Marseille-*P3106*, Clostridium clostridioforme, Clostridiaceae bacterium, Caproiciproducens galactitolivorans, Clostridioides difficile* |
|  | ST11 | Adhesin | WLLKYNNITPFAQKNLVFYDEQVDSR | *Streptococcus* sp. I-P16*, Streptococcus australis, Peptoniphilus lacrimalis* DNF00528 |
|  | ST8 | adhesin P1/ Cell surface antigen I/II | ADYEAKLAKYQADLAK | *Streptococcus mutans, Streptococcus intermedius, Streptococcus anginosus* |
|  | ST7 | CppA protein | NLFQGRENFIPK | *Streptococcus anginosus* |
|  | ST9 | Transposase TcpC | TLEQFLDGYVSRYFTYDSQAGSSDENISK | *Streptococcus pneumoniae, Streptococcus oralis, Streptococcus* sp. HMSC056C01, *Streptococcus* sp. SK140, *Streptococcus infantis* SK1302 |
|  | ST5 | LytR family transcriptional regulator | AHTVQIITEEASFNM*VQNLSNLENQYGETLM*R | *Streptococcus oralis* |
|  | ST14 | General stress protein 17 | M*KPVVKEYTNDEQLM*KDVEELQK | *Bacillus, Bacillus subtilis* |
|  | ST2 | Asp23 protein | SGLSGGFSAVQEKVGEGVEAVKDAASSNENTR | *Streptococcus cristatus* |
|  | ST12 | Asp23protein | KM*TDLDVIEVNVKVVDIK | *Streptococcus phocae, Streptococcus canis, Streptococcus ictaluri, Streptococcus pyogenes, Streptococcus dysgalactiae, Streptococcus dysgalactiae* subsp. *equisimilis, Streptococcus dysgalactiae* subsp. *dysgalactiae, Streptococcus dysgalactiae* subsp. *equisimilis* SK1249 |
|  | ST14 | Asp23 protein | ATEDGSIAVDVYTVLSYGTKISEVSKNIQER | *Streptococcus infantis, Streptococcus oralis, Streptococcus mitis* |
|  | ST2 | Type VII secretion protein EsaA | NSDVSTALSNIWFEAIDSNLKK | *Streptococcus oralis* |
|  | ST2 | Type VII secretion protein EssB | LRLALNLLDLEQALSLPVTFFLHPENLFITK | *Streptococcus pantholopis* |
|  | ST8 | Type VII secretion protein EssB | LEFVREDNQISVQISSSGYRR | *Streptococcus* sp.*,*  *Streptococcus mitis* |
|  | ST14 | Virulence factor | VFGQTDETTIPLLANALADSM*NQSELETLPR | *Streptococcus macedonicus, Streptococcus equinus* |
|  | ST3 | Virulence-associated protein E | M*KATVDNYVLVLRNDPYISESLK | *Streptococcus pasteurianus* |
|  | ST9 | Equibactin | LYEISLKVADC*LGKNGVK | *Streptococcus equi* |
| Antimicrobial production | ST3 | Bacteriocin | WTSKSSKAYAYAGQTSYAFIK | *Streptococcus salivarius* |
|  | ST2 | Bacteriocin | M*SQKIGIM*M*NIK | *Streptococcus intermedius* |
|  | ST14 | Bacteriocin-associated integral membrane protein | AIAVGFSLAGVLAILM*QK | *Streptococcus pneumoniae* |
|  | ST4 | LanT protein | QNVDKLHFTRFDK | *Streptococcus pneumoniae* |
|  | ST12 | LanM protein | RAATKFM*INTDC*PSK | *Streptococcus pneumoniae* |
| ABC transporter | ST2 | Metal ABC transporter | DGADYISVM*QDNLKALEK | *Streptococcus varani* |
|  | ST6 | Metal ABC transporter | VPSAYIWEINTEEEGTPDQISSLIEK | *Streptococcus pyogenes, Streptococcus equi* subsp. *zooepidemicus* Sz105, *Streptococcus canis, Streptococcus castoreus, Streptococcus porcinus, Streptococcus ictaluri, Streptococcus equi* |
|  | ST4 | Nickel ABC transporter | SQPM*NTKM*IVANAGNKDSAVSDK | *Staphylococcus warneri* VCU121, *Staphylococcus warneri, Streptococcus pneumoniae* |
|  | ST10 | Copper ABC transporter | SM*PDAIYLFTLLKVAC*M*GLTSFYSLR | *Streptococcus infantarius, Streptococcus lutetiensis, Streptococcus equinus, Streptococcus* sp. CNU 77-61, *Streptococcus* sp. KCJ4932 |
|  | ST1 | Copper ABC transporter | NNLTLYENQYSLPIAFASQSIYNNVK | *Streptococcus mitis* |
|  | ST10 | Zinc ABC transporter | AVIARM*FASDPNIFVLDEPTTGM*DAGSK | *Streptococcus* spp. |
|  | ST3 | Zinc ABC transporter | TIYKNFM*EIGTAILM*STGLAISLIVM*SKGK | *Streptococcus cristatus, Streptococcus* sp. HMSC062B01, *Streptococcus gordonii,* |
|  | ST2 | Cobalt or another cation ABC transporter | DGKLREVFQIPSYEM*TQVASK | *Streptococcus pneumoniae* |
|  | ST3 | Cobalt ABC transporter | LSSDPVEVTQYYIEKGGPNV | *Streptococcus salivarius* |
|  | ST2 | Cobalt ABC transporter (CbiM) | IISKDPNSKTM*LALSGAFIFILSSLK | *Streptococcus australis, Streptococcus parasanguinis* |
|  | ST4 | FeoABC transporter (FeoB) | LM*DM*GLTHHTKIYLRK | *Streptococcus gallolyticus* |
|  | ST10 | FeoABC transporter (FeoB) | RNLQLTIQLLELNVPVM*IGLNM*IDVSAK | *Staphylococcus warneri, Staphylococcus epidermidis, Mycobacteroides abscessus* subsp. *abscessus* |
|  | ST9 | FeoABC transporter (FeoB) | EATGNQNISPNLTISNAQLNLEDKNK | *Streptococcus dysgalactiae* |
|  | ST1 | Bacitracin ABC transporter (BceAB) | TVLGFGC*FVVQLVVIILVAYANGYVM*K | *Streptococcus* sp. HSISM1, *Streptococcus parasanguinis* |
|  | ST14 | Bacitracin ABC transporter (BceAB) | QNIIALIQENGIKKSVLAK | *Streptococcus* sp. SK643, *Streptococcus pseudopneumoniae* |
|  | ST2 | Bacitracin ABC transporter | SVEYPEKIATLLVNAGYPPK | *Streptococcus sanguinis* |
|  | ST9 and ST12 | Bacteriocin ABC transporter | VNKGEFIAIM*GESGSGK | *Streptococcus phocae* |
|  | ST9 | Bacteriocin ABC transporter | M*IVNFYTPNHGQITLGDYDLK | *Streptococcus gallolyticus* |
|  | ST4 | Bacteriocin ABC transporter | KTVEDLSM*M*KGDM*TFK | *Streptococcus oralis, Streptococcus* sp. NPS 308, *Streptococcus* sp. oral taxon 071 str. 73H25AP, *Streptococcus mitis, Streptococcus* sp. VT 162, *Streptococcus australis, Streptococcus pseudopneumoniae, Streptococcus halitosis, Streptococcus* spp. |
|  | ST9 | Lantibiotic Mutacin ABC transporter protein (MutE) | LM*VPILNILPNGLPAGTDAVVAPK | *Streptococcus sobrinus* |
|  | ST4 | Lantibiotic ABC transporter | STIM*KIIFGLENADSGAIVFNGGKNAGK | *Streptococcus mitis* |
|  | ST2 | Amino acid ABC transporter | TIDLSQPITTETLLWVR | *Weissella confusa* |
|  | ST2 | Amino acid ABC transporter | QVLFTKPYM*ANKQVLVTKK | *Floricoccus penangensis* |
|  | ST14 | Amino acid ABC transporter | M*VDGKNQVVGADIGM*AQAIADELGVK | *Streptococcus oralis* |
|  | ST5 | Amino acid ABC transporter | LINFAHGDIYM*VGAFM*GYFLLNSLK | *Streptococcus australis* ATCC 700641*, Streptococcaceae bacterium, Streptococcus parasanguinis, Mycobacterium tuberculosis,* |
|  | ST3 | Amino acid ABC transporter | NLTDKSQM*NIGIFFAIIALVVIWFLM*KK | *Streptococcus parasanguinis* |
|  | ST13 | Amino acid ABC transporter | TGVPLLTPSGTQDDLTVDAK | *Streptococcus* sp. 449_SSPC, *Streptococcus salivarius,* |
|  | ST14 | Amino acid ABC transporter | VIFM*DKGIIAEEGKPEDLFTNPKEER | *Streptococcus* sp. oral taxon 058, *Streptococcus oralis* |
|  | ST13 | Amino acid ABC transporter | IVLPQAFRIALPNLTTALLNLM*R | *Streptococcus* sp. AS14, *Streptococcus sanguinis, Streptococcus cristatus, Streptococcus* sp. *CCH8-C6* |
|  | ST9, ST13 and ST14 | Amino acid ABC transporter | NLLLAPVKVQKR | *Streptococcus* sp. 45, *Streptococcus infantarius, Streptococcus* sp. KCJ4932, *Streptococcus infantarius sub*sp. *infantarius* CJ18*,*  *Streptococcus lutetiensis* 033, *Streptococcus infantarius, Streptococcus equinus* |
|  | ST10 | Glutamine ABC transporter | DASLAPM*FVAGAIYLIM*IGLVTLISKQVEK | *Streptococcus* sp. DD13 |
|  | ST13 | Glutamine ABC transporter | KDEVIKEAENLLER | *Streptococcus sanguinis* |
|  | ST14 | Glycine/betaine ABC transporter | YDLQVLEDDKQLFPPYQGAPLM*KEDLLK | *Streptococcus oralis, Streptococcus mitis* |
|  | ST2 | Glycine/betaine ABC transporter | QEITLAYVEWDSEVASTNVLAEVLKTK | *Streptococcus infantarius* |
|  | ST4 | Glycine/betaine ABC transporter | AKLRTIVAAFAVM*VLGLGASYAPSM*IPSK | *Streptococcus infantis* |
|  | ST14 | Oligopeptide ABC transporter | KNVQM*IFQDPQASLNAR | *Streptococcus infantarius, Streptococcus lutetiensis* |
|  | ST1 | Multidrug ABC transporter | QLQQYIYESLLTTSVK | *Streptococcus suis* |
|  | ST4 | Multidrug ABC transporter | SGSKALKQLQQYIYESLLTTSVK | *Streptococcus suis* |
|  | ST10 | Multidrug ABC transporter | LESKEIDENSIVSK | *Streptococcus pneumoniae, Streptococcus salivarius, Streptococcus* sp. HMSC068F04, *Streptococcus* sp. FDAARGOS_192, *Streptococcus* sp. SR4, *Streptococcus thermophilus, Streptococcus* sp. C150, *Streptococcus* sp. HMSC064H09,  *Streptococcus* sp. HMSC064H03, *Streptococcus* sp. HSISS2 |
|  | ST2 | Multidrug ABC transporter | AQGTLADLQATFGDASASLNDIYLALTKEV | *Streptococcus phocae* |
|  | ST1 | Multidrug ABC transporter | YLLNLDEKQINIAPHLTINHLK | *Streptococcus* |
|  | ST1 | Multidrug ABC transporter | M*PTAFYLFFSSM*YQDTPGGPANFM*R | *Streptococcus pneumoniae* |
|  | ST5 | Multidrug ABC transporter | TTLIM*VSQRTNSLAK | *Streptococcus* sp. *caviae* |
|  | ST7 | Multidrug ABC transporter | FPNAFYLSM*SILLVQAVLNM*R | *Streptococcus pantholopis* |
|  | ST3 | Multidrug ABC transporter | SGVVLSLLGAM*ISFILYLVFLKANIK | *Streptococcus* sp. HMSC066E07, *Streptococcus anginosus* |
|  | ST13 | Choline ABC transporter | IIIAIASM*LQTIPSLALLALM*IPLFGIGK | *Streptococcus agalactiae,*  *Listeria monocytogenes, Streptococcus agalactiae* |
|  | ST7 | Multidrug ABC transporter | IAYLPQEGALFHDTVLYNLTIGREVPEDR | *Streptococcus suis* |
|  | ST14 | Macrolide ABC transporter (MacB) | STLM*NIIGM*LDRPTSGEYYLEGEEVAKLSEK 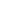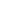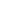 | *Streptococcus anginosus, Streptococcus* sp. KCOM 2412, *Streptococcus* sp. HMSC057E02 |
| Other Transporters | ST8 | Manganese transport protein MntH | GKEFLPFVNHSAIAGILTTGVM*R | *Staphylococcus warneri* |
|  | ST2 | Manganese transport protein MntH | YLLLSVVLISSLIAM*QLQQM*AGKLGIVTQK | *Streptococcus equinus, Streptococcus* sp. KCJ4950 |
|  | ST6 | HlyC/CorC family transporter | TAPVIIFLGKIVSPFVWLLSASTNLLSQM*TPM*K | *Streptococcus cristatus, Streptococcus* sp. *marseille-*P644, *Streptococcus* sp. *marseille-*P7375 |
|  | ST4 | HlyC/CorC family transporter | ISNYIHELPM*ISETTR | *Staphylococcus* spp. |
|  | ST2 and ST8 | HlyC/CorC family transporter | DEIIGM*VNVKDLFIR | *Bacillus* spp. |
|  | ST4 | Metal-binding protein | KAILQLETLAC*PTC*M*QK | *Streptococcus gallolyticus, Streptococcus macedonicus, Streptococcus infantarius, Streptomyces xinghaiensis, Lactobacillus delbrueckii, Enterococcus cecorum, Lactobacillus delbrueckii, Aeriscardovia aeriphila, Lactobacillus porci,*  *Lactobacillus agilis, Bifidobacterium pseudolongum, Enterococcus cecorum.* |
|  | ST4 | Multidrug MFS transporter | M*VIVLC*SILIAIVVLGAFVFPVK | *Streptococcus agalactiae, Enterococcus faecalis* |
|  | ST2 | multidrug efflux MFS transporter (NorA) | ILGGFSAGM*VM*PGVTGM*IADISK | *Bacilli, Staphylococcus* spp.*, Staphylococcus hominis* |
|  | ST9 | Multidrug transporter MatE | AM*LIM*SLGAGINIVLDPVLM*IM*FK | *Streptococcus intermedius, Streptococcus* sp. AS20 |
|  | ST14 | MFS Lantibiotic transporter | DLWC*NM*IIAAK | *Streptococcus dysgalactiae* |

(M* methionine oxidation; C* carbamidomethylation of Cys).
